# Insights into the evolution and mechanisms of response to heat stress by whole genome sequencing and comparative proteomics analysis of the domesticated edible mushroom *Lepista sordida*

**DOI:** 10.1080/21501203.2024.2363620

**Published:** 2024-07-07

**Authors:** Yanjiao Zhang, Chengzhi Mao, Xuyang Liu, Lizhong Guo, Chunhui Hu, Xiaobo Li, Lili Xu, Hao Yu

**Affiliations:** a Shandong Provincial Key Laboratory of Applied Mycology, School of Life Sciences, Qingdao Agricultural University, Qingdao, China; b Shandong Mushroom Industrial Technology Innovation Research Institute, Jining, China

**Keywords:** Edible mushroom, *Lepista sordida*, genome, proteome, heat stress response

## Abstract

*Lepista sordida* is a valuable edible mushroom rich in natural bioactive compounds. In the present study, a high-quality whole-genome of a domesticated strain of *L. sordida* was sequenced, revealing a 40.67 Mb genome in 13 contigs. Phylogenetic analysis revealed that *L. sordida* is evolutionarily closely related to edible mushroom *Lyophyllum decastes* and *Hypsizygus marmoreus*. Heat stress has a significant effect on the yield and quality of mushrooms, but the molecular basis for this is poorly understood in *L. sordida*. A label-free comparative proteomic analysis was performed under different heat stress conditions. The growth of *L. sordida* mycelia was inhibited, and nuclear apoptosis occurred under heat stress. Ca^2+^ and MAPK signaling pathways were found to be involved in heat stress signal transduction. It is hypothesized that the expression of various heat shock proteins plays a crucial role in the response to heat stress. In addition, the components of the ubiquitin-proteasome system and the thioredoxin system were upregulated, preventing the accumulation of misfolded proteins and possibly supporting the response to heat stress. In summary, these results provide a fundamental insight into the evolution and heat stress-responsive mechanisms in *L. sordida* and may facilitate the breeding of heat-tolerant strains for artificial cultivation.

## 1. Introduction

*Lepista sordida* is a delicious edible mushroom that is widely distributed in the northern temperate zones of the world (Choi et al. 2010a, 2010b). In China, it is known by various names, such as “Hualianxiangmo” due to its bright colouration and excellent flavour, and it is also called “Dingxiangmo” and “Hualianmo” due to its striking deep lilac or purple pileus colour (Li et al. 2014; Sheng et al. 2020; Zhao et al. 2022). Its fruiting body is rich in essential nutrients and contains 18 types of amino acids, including 8 important for human health, as well as zinc, iron, and other vital trace elements (Meng et al. 2016). Moreove, *L. sordida* has been shown to possess multiple bioactive metabolites, such as polysaccharides, diterpenoids, deoxygenated diketopiperazines, sesquiterpenes, lepistamides A–C, and diatretol. These compounds have immunoregulatory (Luo et al. 2012), antioxidant (Zhong et al. 2013; Wang et al. 2016; Acharya et al. 2019; Aranha et al. 2022), antitumor activities (Miao et al. 2013a, 2013b; Hu et al. 2016), anti-ageing activities (Zhong et al. 2013), antibacterial, and antifungal activities (Mazur et al. 1996; Acharya et al. 2019). These studies have shown that *L. sordida* is a valuable functional food.

The wild strains of *L. sordida* have been successfully domesticated and cultivated artificially since 2000 (Tian et al. 2003). Most of the research has focused on bioactive compounds and cultivation conditions of *L. sordida* strain (Mazur et al. 1996; Alves et al. 2012; Luo et al. 2012; Miao et al. 2013a, 2013b; Zhong et al. 2013; Hu et al. 2016; Wang et al. 2016; Acharya et al. 2019; Wang et al. 2023). However, to date, *L. sordida* has not been extensively cultured due to the lack of high-quality strains of *L. sordida*. A previous study investigated the mechanisms underlying *L. sordida* development and identified the key pathways involved in primordia formation and fruiting body development (in press).

emperature plays a crucial role in the growth of edible mushrooms and the development of fruiting bodies (Xu et al. 2021; Xie et al. 2024), especially for straw-rotting mushrooms. High temperatures in composts and growing environments are among the main factors contributing to production losses. The strain of *L. sordida* is a fruiting mushroom with moderate temperatures. The mycelia grow vegetatively at 23 °C to 30 °C and transform into the fruiting process at around 25 °C (Li et al. 2014; Thongbai et al. 2017). High temperature is a significant unfavourable abiotic stressor that affects various biological tissues, including growth and development, at different levels, inhibits mycelial growth, causes poor fruiting body development, triggers autolysis or apoptosis of cells, and affects the synthesis of secondary metabolites (Xu et al. 2021; Xie et al. 2024). However, to date, little has been reported on the response to heat stress in strain *L. sordida*. High temperatures above 34 °C can inhibit the mycelial growth of strain *L. sordida* (Xie and Hu 2005; Zhang et al. 2006). Zheng et al. (2011) reported that the high temperatures in the autumn of 2010 led to the extinction of wild strains of *L. sordida* in the Sandaoguan Nature Reserve. Mycelial catalase activity, H_2_O_2_ content and expression of identified catalase genes in strain *L. sordida* were affected under heat stress of 35 °C (Wang et al. 2022). Further studies on the regulatory mechanism of *L. sordida* under heat stress are needed.

High quality genome sequences are the basis for the genetic research and molecular breeding of mushrooms (Dong et al. 2022). The genome of a wild *L. sordida* strain was recently sequenced (Takano et al. 2019) with illumina platform. However, high quality genome sequences of *L. sordida* have not yet been reported to date and no genome of a domesticated *L. sordida* strain has been published. In the present study, the high quality genomes of *L. sordida* were sequenced using Illumina and Nanopore sequencing platforms. The lignocellulose-degrading enzymes were analysed to provide useful information for substrate optimisation experiments. In addition, the protein expression profiles of *L. sordida* at normal temperature, elevated temperature and in the recovery phase after heat stress were investigated to clarify the adaption mechanism. The genome assembly and proteome analysis of *L. sordida* will facilitate the breeding of high-quality, high-yielding strains with specific local traits and thus enable extensive cultivation.

## 2. Materials and methods

### 2.1. Samples and cultivation

**Figure 1.**
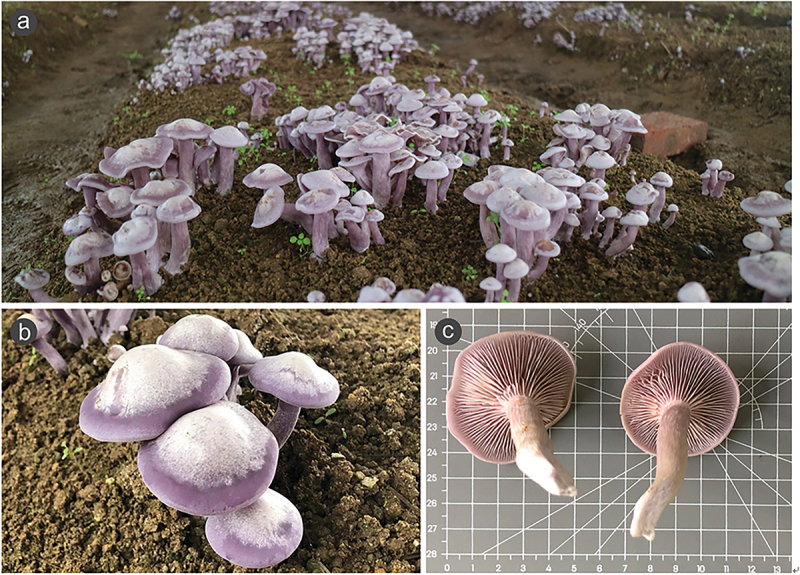
The fruiting bodies of *Lepista sordida*. (a) The morphological characteristics of the fruiting body of *L.* *sordida*. (b) and (c) *L. sordida* were cultivated in greenhouse.

The dikaryon strain *L. sordida* WT2342 (formerly known as *L. sordida* MCCWT230001) used in this study was provided by the Laboratory of Mushroom Precision Breeding (http://mushroomlab.cn/). Strain WT2342 was domesticated from wild fruiting bodies collected in the Inner Mongolia Autonomous Region of China (Figure 1). Two monokaryotic strains, namely Lds1 and Lds5, were isolated from the spores of strain WT2342 using the standard dilution plating technique as described previously (Xu et al. 2023). Unless otherwise indicated, mycelia of *L. sordida* strains were routinely cultured and preserved on YMA plates (extract of 200 g potato, 20 g glucose, 3 g yeast extract, 0.5 g MgSO_4_, 20 g agar powder, and 1 L deionised water) at 25 °C in the dark. In addition, coverslips were used along the edge of the medium to facilitate the collection of mycelia for micromorphological examination.

### 2.2. Genome sequencing and assembling

The *L. sordida* strains were cultivated on YMA plates covered with cellophane. Once the plates were fully colonised, the mycelia were scraped from the cellophane, rapidly frozen in liquid nitrogen and then stored at −80 °C for subsequent analyses. These analyses were performed at Benagen Technology Co., Ltd. (Wuhan, China). Genome sequencing and RNA-Seq were performed according to the previously described method, with slight adaptations (Zhu et al. 2023). In summary, genomic DNA was extracted using the NucleoBond HMW DNA kit (Macherey-Nagel, Germany) and sequenced using the Illumina NovaSeq 6000 platform (paired-end, 2 × 150 bp) and the Nanopore PromethION 48 sequencing platform, achieving a sequencing depth of > 50× and > 100×, respectively. The RNA was extracted with Trizol (TaKaRa, China) and sequenced on the Illumina NovaSeq 6000 sequencing platform.

The genome assembly process followed the previously described (Xu et al. 2023). Briefly, the genome was assembled using Necat software with default parameters with some modifications GENOME_SIZE = 40000000, PREP_OUTPUT_COVERAGE = 80, and CNS_OUTPUT_COVERAGE = 80. In brief, Nanopore sequencing reads were initially filtered using Filtlong software, and then assembled using NECAT tools with default parameters (Chen et al. 2021). Following this, the resultant assembly underwent two rounds of polishing using Racon with Nanopore reads. Subsequently, the refined contigs were further polished through two iterations using filtered Illumina reads via Pilon.

### 2.3. Gene prediction and annotation

Gene predictions were derived from three different methods as previously described (Xu et al. 2023): homology-based prediction using Augustus software (Stanke et al. 2006), *ab initio*-based prediction using Genemark-ES, and gene prediction was also performed based on RNA-Seq transcripts using StringTie software. The three predicted genes were integrated using EVidenceModeler software (Brini 2009). The repeat sequence was analysed using the software RepeatModeler and RepeatMasker (Chen 2004; Flynn et al. 2020). The completeness of gene prediction was assessed using BUSCO software with fungi_odb10. The functional annotations of the proteins were performed using eggNGOmapper software, the Pfam database and the SwissProt databases (Yu et al. 2022). In addition, the annotation of CAZymes was performed using the software Dbcan version v3.0.2 with Hmmer search engine and default parameters (Yu et al. 2022). Circular layouts were created using the Circos software (http://circos.ca/, accessed 20 December 2022) (Krzywinski et al. 2009).

### 2.4. Genome evolutionary and comparative analysis

Pairwise average nucleotide identity (ANI) analysis was performed using FastANI software (Jain et al. 2018). Collinearity analysis was performed using TBtools software (Chen et al. 2020). Gene families and orthogroups were analysed with the software OrthoFinder (Emms and Kelly 2019). The phylogenomic tree was constructed by concatenating single-copy orthologous protein sequences and visualised using FastTree software (Price et al. 2009). Divergence time analysis was performed using Bayesian software package MCMCtree using the reference divergence time between *Lentinula edodes* and *Saccharomyces cerevisiae* (626–806 MYA) and the divergence time between *L. edodes* and *Agaricus bisporus* (130–185 MYA) from the Timetree database (http://timetree.org). The *in silico* DNA-DNA hybridisation (*is*DDH) values were estimated with the Genome-to-Genome Distance Calculator 3.0 (GGDC) using the recommended formula 2 (Meier-Kolthoff et al. 2022).

### 2.5. *Heat treatment of* L. sordida *mycelia for proteomic analysis*

In this study, the dikaryotic mycelium of strain WT2342 was cultivated and analysed for its response to heat stress. The mycelia were cultured on YMA plates with cellophane in darkness. The mycelia cultured at 25 °C for 16 days were collected as the Con group. In addition, the mycelia cultured at 25 °C for 15 days and then transferred to 37 °C for 8 hours were collected as the HS8 group. Mycelia cultured at 25 °C for 15 days and then transferred to 37 °C for 24 hours were collected as the HS24 group. Another group of mycelia cultured at 25 °C for 11 days, exposed to 37 °C for 24 hours and then returned to 25 °C for 7 days was collected as the RC group (Supplementary Figure S1). All samples were immediately frozen in liquid nitrogen and stored at −80 °C for subsequent protein extraction. Each group consisted of three biological replicates.

### 2.6. Protein extraction, trypsin digestion, and LC-MS/MS analysis

Total mycelial protein was extracted as in our previous description (Xu et al. 2021; Xie et al. 2024) with slight modifications. The mycelia suspended in UA buffer (8 mol/L urea, 0.1 mol/L pH 8.5 Tris-HCl) with phenylmethylsulfonyl fluoride (PMSF) and Halt^TM^ Protease Inhibitor Cocktail (Thermo Fisher Scientific) were disrupted using the TissueLyser II and ultrasonication, and then centrifuged to remove cell debris. The protein concentration in the resulting supernatant was measured using a Micro BCA Protein Assay Kit (Thermo Fisher Scientific). For trypsin digestion, proteins were reduced with dithiothreitol (DTT) and alkylated with iodoacetamide (IAA). Digestion was performed using the STrap method as previously described by us (Xu et al. 2023). Post-digestion, the peptides were desalted using a C18 column. The peptide content was measured using the Pierce^TM^ Quantitative Colorimetric Peptide Assay (Thermo Fisher Scientific) and the peptides were lyophilised.

The desalted peptides were dissolved in formic acid (0.1%). Label-free analysis was performed using a Nano-LC system coupled to an Orbitrap Fusion Tribrid (Thermo Fisher Scientific) operating in data-dependent acquisition (DDA) mode. Full MS scans over a mass range of m/z 350–1,500 with detection in the Orbitrap (120 K resolution) and with auto gain control set to 100,000. Different chromatographic gradient lengths from 60 to 240 min were tested for peptide separation. The gradient started at 5% (v/v) ACN (0.1% formic acid) and went up to 32% (v/v) ACN (0.1% formic acid). Each group included three biological replicates.

Protein identification and quantification were performed using Proteome Discoverer 2.2 against the *L. sordida* proteome. Sample abundances were normalised to an identical sum by standardisation. Differentially expressed proteins (DEPs) were identified using the following criteria: *p*-value < 0.05 and fold-change > 2.

### 2.7. Bioinformatic analysis

Venn plot, volcano plot, heat map, and PCA analysis were performed and visualised with Venn, ggplot2, factoextra, and FactoMineR. The Gene Ontology (GO) [http://geneontology.org/ (accessed on 3 April 2022)] and the Kyoto Encyclopedia of Genes and Genomes (KEGG) [http://www.kegg.jp/ (accessed on 7 December 2023)] were used to analyse the functions and metabolic pathways of the differentially abundant proteins. The results of the GO analysis were visualised with TBtools.

## 3. Results

### 3.1. *Genome assembly of monokaryotic* L. sordida

*Lepista* *sordida* WT2342 was a wild mushroom species that was successfully domesticated and cultivated by our laboratory in recent years (Figure 1). In order to decipher its genome information for genetic breeding, the genome of the monokaryotic strains Lds1 and Lds5 derived from strain WT2342 was sequenced. For strain Lds1, a total of 6.34 Gb and 5.65 Gb of data were obtained from Nanopore and Illumina, respectively. After filtering, the clean data were assembled with Necat and polished with Racon and Pilon. Finally, 13 contigs with an N50 of 3.85 Mb and an N90 of 2.51 Mb were obtained after the repeats were removed. The total genome length is 40.67 Mb and the length of the largest contig is 7.73 Mb (Table 1). The GC content of *L. sordida* is 46.35%, which is similar to that of strain *L. sordida* NBRC 112841 (Takano et al. 2019). Repeat sequences accounted for 15.24% of the whole genome of Lds1 and the majority were LTR elements (11.91%), DNA transposons (0.75%), the LINE (0.52%), and simple repeats (0.43%) (Table 2). GenomeScope was used to generate a histogram of the sequencing depth distribution (k = 19) (Supplementary Figure S2). A single *k*-mer coverage peak was observed and the heterozygosity rate was 0.03%. These results confirmed the monokaryotic nature of strain Lds1. The genome information of strain Lds5 is shown in Supplementary Table S1.

**Table 1.** Genome assembly features of monokaryotic *Lepista sordida* Lds1.

Characteristics	*L. sordida* Lds5
Genome assembly size (Mb)	40.67
Contigs	13
Longest Contigs (kb)	7,731.9
Contigs N50 (kb)	3,848.6
Contigs N90 (kb)	2,514.9
GC (%)	46.35
Sequencing methods	Nanopore; Illumina

**Table 2.** Repeat element analysis in the genome of *Lepista sordida*.

Repeat elements	Copies (numbers)	Repeat size (bp)	Percentage of the assembled genome (%)
LINEs	140	211,346	0.52
LTR/Copia	363	489,404	1.20
LTR/Gypsy	1,740	3,623,196	8.91
LTR/Retroviral	60	19,420	0.05
DNA transposons/hobo-Activator	157	180,081	0.44
DNA transposons/Tc1-IS630-Pogo	28	10,146	0.02
Tourist/Harbinger	6	9,826	0.02
Rolling-circles	74	89,097	0.22
Unclassified	3,245	1,363,819	3.35
Simple repeats	3,824	175,122	0.43
Low complexity	621	33,136	0.08
Total	10,258	5,503,843	15.24

### 3.2. *Gene prediction and annotation of* L. sordida

A total of 12,222 coding sequences (CDSs) with an average length of 1,711 base pairs were predicted in the genome of *L. sordida*. The cumulative length of these coding sequences was 20.91 Mb, accounting for 51.42% of the entire genome. The average number of exons and introns per gene was 6.8 and 5.8, respectively (Table 3). The completeness of genome assembly and gene prediction of strain Lds1 was assessed using the BUSCO software. The Fungi_odb10 database (758 genes) was analysed with a completeness of 95.9%, the Basidiomycota_odb10 database (1,764 genes) with a completeness of 93.9%, and the Agaricales_odb10 database (3,870 genes) with a completeness of 90.1% (Supplementary Figure S3). These results indicate that the genome sequence of the monokaryotic strain Lds1 is of high quality and has high integrity and continuity.

**Table 3.** Characteristics of the gene prediction of *Lepista sordida* Lds1.

Characteristics	Number/length
CDSs numbers	12,222
Total concatenated gene length (bp)	26,094,780.95
Average gene length (bp)	2,135.07
Total exon number (bp)	82,905
Total exon length (bp)	20,831,324.69
Average exon length (bp)	251.27
Average exon number	6.78
Total intron number	70,863
Average intron number	5.78
Total intron length (bp)	5,135,893
Average intron length (bp)	72.48

**Figure 2.**
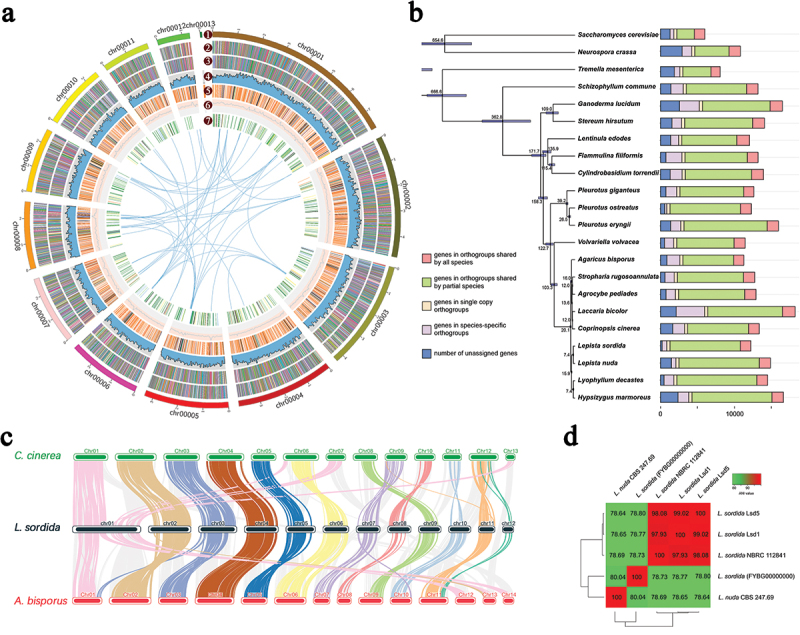
Genome information of *Lepista sordida* Lds1. (a) Overview of the *L. sordida* genome assembly and gene prediction. A total of seven layers were applied from outside to inside. The outermost layer is a circular representation of the 13 contigs with size intervals of 1 Mb. Layers two and three represent the predicted genes in the forward and reverse strands of the genome. Layer four shows the gene density. Layer five shows the repeat sequences. Layer six shows the GC content. Layer seven shows the genes of CAZymes. (b) Phylogenetic tree based on 433 single-copy orthologs from *L. sordida* and 21 other fungal species. The number of different orthologous gene types was calculated for each fungal species and marked with different colors. (c) The collinearity analysis of *L. sordida*, *Agaricus bisporus*, and *Coprinopsis cinerea*. (d) Cluster heatmap of average nucleotide identity (ANI) values between *L. sordida* Lds1, Lds5, and the other three strains.

The predicted gene sequence was functionally analysed using the EggNOGmapper, SwissProt, and Pfam databases. Within the annotated genes 10,765 and 6,407 genes were classified by the EggNOGmapper and SwissProt databases, respectively. In addition, a total of 8,463 genes were annotated from the Pfam database via Hmmer based on protein domain similarity. Finally, all genomic information of strain Lds1 was visualised in a circular mapping based on the results of genome assembly and functional annotation using Circos software (Figure 2a).

### 3.3. Genome evolutionary and comparative analysis

The genus *Lepista* is polyphyletic and comprises over 50 species. Due to the lack of genomic data, the genetic placement of *L. sordida* within its genus and among closely related species of other genera remains unclear (Thongbai et al. 2017). Therefore, phylogenetic analyses were conducted to investigate the evolutionary relationships between *L. sordida* and 21 other fungal species. The reconstruction of the phylogenomic tree and estimation of the divergence time of the species were performed based on 433 single-copy orthologous genes (Figure 2). The phylogenomic tree (Figure 2b) showed that strains *L. sordida* and *L. nuda* formed a distinct phylogenetic clade within the genus *Lepista*, confirming their closest phylogenetic relationship. Furthermore, *L. sordida* was shown to be evolutionarily closely related to *Lyophyllum decastes* and *Hypsizygus marmoreus*. It was estimated that the divergence between the species of *L. sordida* and *L. nuda* occurred about 7.41 million years ago (MYA). *L. sordida* split from *H. marmoreus* around 16.0 MYA. *L. sordida* and *A. bisporus* had a common ancestor about 20.2 MYA ago (Figure 2b). The number of different orthologous gene types was calculated for each fungal species and marked with different colours (Figure 2b).

Collinearity analysis was performed between *L. sordida* and two other edible mushrooms, *A. bisporus* and *Coprinopsis cinerea*, which have genome assemblies at the chromosome level, using TBtools software (Figure 2c). According to the collinearity results, significant chromosomal ruptures and fusion events were detected in contig 1 and contig 11 of *L. sordida* when compared to *C. cinerea*. Moreover, rupture and fusion events were observed in Contig 1, Contig 2, Contig 3, Contig 7, Contig 11, and Contig 12 of *L. sordida* compared to the chromosomes of *A. bisporu*s. The analysis revealed more rupture and fusion events between *L. sordida* and *A. bisporus* than between *L. sordida* and *C. cinerea*, which is consistent with the phylogenetic tree analysis. Specifically, contig 1 of *L. sordida* fragmented into four chromosomes (chr1, chr6, chr7, and chr13) in *C. cinerea* and four chromosomes (chr1, chr12, chr13, and chr14) in *A. bisporus*. In addition, Contig 11 and Contig 12 could belong to the same chromosome. Furthermore, only Contig 13 showed no similarity with the genome of the other two strains. These results demonstrate the high quality of the *L. sordida* genome assembly.

Average nucleotide identity (ANI) analysis was performed to estimate the genomic differences and relatedness between the genomes of *Lepista* strains. Two genomes of *L. sordida* strains [*L. sordida* (FYBG00000000) and *L. sordida* NBRC 112841] and strain *L. nuda* CBS 247.69 were obtained from the NCBI genome database to compare with the two monokaryotic *L. sordida* genomes, Lds1 and Lds5, assembled in this study. As shown in Figure 2d, the ANI percentages between the monokaryotic mycelia Lds1 and Lds5, which are derived from the same dikaryotic strain *L. sordida* WT2342 were 99.02%. The ANI values between *L. sordida* Lds1 and three other strains [*L. sordida* NBRC 112841, *L. sordida* (FYBG00000000), and *L. nuda* CBS 247.69] were 97.93%, 78.77%, and 78.65%, respectively. The ANI percentages between the strains (*L. sordida* Lds1, Lds5, and *L. sordida* NBRC 112841) were all above 97.93%, exceeding the threshold for ANI (95%–96%) typically used to distinguish bacterial species (Chun et al. 2018). Furthermore, the ANI percentages between *Lepista nuda* CBS 247.69 and other strains within the species *L. sordida* were all below 80.05%. It is worth noting that the ANI percentages between *L. sordida* (FYBG00000000) and the other strains were all below 80.05%.

The *is*DDH percentages between monokaryotic mycelia Lds1 and Lds5 derived from the same dikaryotic strain *L. sordida* WT2342 were 90.80% (Table 4). The *is*DDH percentages between Lds1, Lds5, and *L. sordida* NBRC 112841 exceed 70%, indicating greater similarity between *L. sordida* WT2342 and *L. sordida* NBRC 112841. Conversely, the *is*DDH percentages between strain *L. nuda* CBS 247.69 and other strains are consistently below 21%. Similarly, strain *L*. *sordida* (FYBG00000000) also shows *is*DDH percentages of less than 21% compared to the other strains. From the above results, it can be concluded that *L. sordida* (FYBG00000000) may not belong to the species *L. sordida*. The taxonomic classification and affiliation of strain *L. sordida* (FYBG00000000) requires further investigation.

**Table 4.** The *in silico* DNA-DNA hybridisation (*is*DDH) values between *Lepista sordida* Lds1, Lds5, and the other three strains.

*is*DDH	*L. sordida* Lds1	*L. sordida* Lds5	*L. sordida* NBRC 112841	*L. sordida* (FYBG00000000)	*L. nuda* CBS 247.69
*L. sordida* Lds1	100.00%	90.80%	76.50%	19.00%	18.70%
*L. sordida* Lds5	90.80%	100.00%	78.80%	19.00%	18.70%
*L. sordida* NBRC 112841	76.50%	78.80%	100.00%	19.00%	18.70%
*L. sordida* (FYBG00000000)	19.00%	19.00%	19.00%	100.00%	20.50%
*L. nuda* CBS 247.69	18.70%	18.70%	18.70%	20.50%	100.00%

Mating-type locus in *L. sordida* were identified by using the mating-type genes from another mushroom as query sequences. Three homeodomain genes and four pheromone receptor genes were identified in contig002 and contig007, respectively (Supplementary Figure S4). The information of mating-type locus could be used for the development of molecular markers for crossbreeding of *L. sordida*.

### 3.4. *Carbohydrate active enzymes (CAZymes) in* L. sordida *genome*

**Figure 3.**
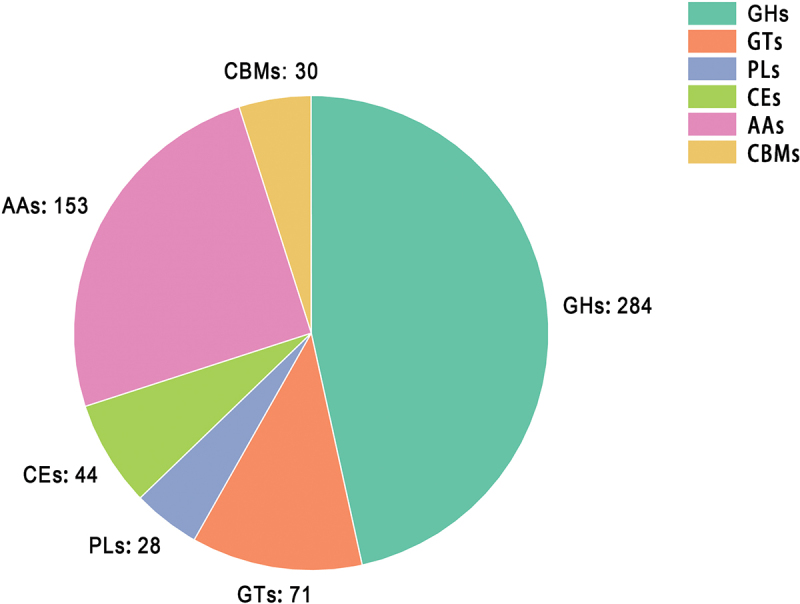
The distribution of carbohydrate-active enzymes CAZymes categories in *Lepista sordida*.

Based on genome annotation and dbCAN analysis, the genome of strain Lds1 encodes 284 glycoside hydrolases (GHs), 71 glycosyl transferases (GTs), 28 polysaccharide lyases (PLs), 44 carbohydrate esterases (CEs), 153 auxiliary activities enzymes (AAs), and 30 carbohydrate-binding modules (CBMs), distributed across 58, 26, 9, 8, 12, and 5 families, respectively (Figure 3). The results showed that the genes in the glycoside hydrolase family were significantly enriched.

GHs accounted for 45.56% of the total CAZymes identified in strain Lds1. In particular, 25 genes were identified in GH5 and 5 genes in GH7, all of which are associated with cellulose digestion. Moreover, 24 GH16 genes, 19 GH18 genes, 12 GH3 genes, 20 GH43 genes, and 8 GH10 genes were also identified, and these genes were also involved in hemicellulose digestion (Li et al. 2022; Zhu et al. 2023). In addition, the proteins within the AA category (25.08%) were diversified across AA3, AA7, AA9, AA1, AA5, and AA2. The AA1 and AA2 categories include laccases and peroxidases, respectively, two types of lignin-degrading enzymes (Li et al. 2022). These results indicate that strain Lds1 is capable of efficiently degrading complex carbohydrates, diversifying its nutrient substrate utilisation and facilitating growth.

### 3.5. *Response to heat stress of strain* L. sordida

The growth rates of *L. sordida* mycelia on YMA plates at different temperatures (25, 30, 34, 37 and 42 °C) were measured to determine the inhibitory effect of high temperatures. The mycelial plugs (9 mm diam.) of *L. sordida* were inoculated in the centre of YMA plates (7 cm diam.) and the mycelia were cultured in the dark for 15 days. The mycelia of strain *L. sordida* showed robust growth at 25 °C and 30 °C. However, after 15 days of cultivation under conditions of 34 °C, 37 °C, and 42 °C, no growth of mycelia was observed (Supplementary Figure S5). The results indicate that temperatures above 34 °C significantly inhibit the growth of strain *L. sordida*.

**Figure 4.**
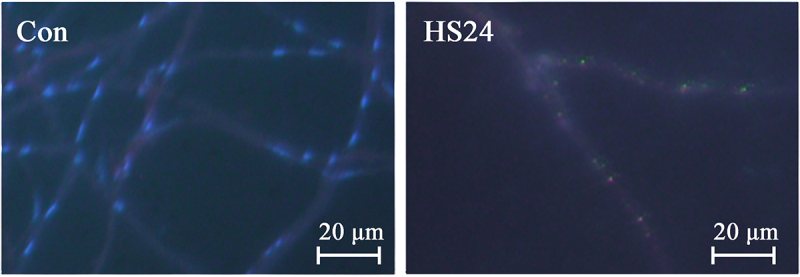
The mycelial morphology observed using positive fluorescence microscope after strained with Congo red (1%, m/v) and DAPI (10 μg/mL, Solarbio). Red, strained cell wall; Green, strained DNA. Microscopic image of *Lepista sordida* mycelia.

Most studies use 37 °C for heat stress response studies. Therefore we investigated the effect of this temperature on the recovery growth and microscopic morphology of the mycelium of *L. sordida*. The mycelial morphology of the control group (Con) and the heat-treated group (HS24) (Supplementary Figure S1) was observed with a positive fluorescence microscope (Axio Scope A1, Germany) in the experimental platform of the School of Life Science at Qingdao Agricultural University after strained with Congo red (1%, m/v) and DAPI (10 μg/mL, Solarbio). As shown in Figure 4, the mycelia in the Con group appeared clear with intact large nuclei. However, after a 24-hour treatment at 37 °C (HS24 group), the mycelia appeared indistinct and the nuclei were fragmented and dispersed throughout the mycelial cells. After the heat stress treatment, the *L. sordida* mycelia were able to resume growth indicating that the damage caused by 37 °C treatment for 24 hours was not an irreversible lethal condition. The mycelia were able to counteract such adverse conditions by regulating gene and protein expressions and metabolic activities.

### 3.6. *Proteomic analysis revealing heat stress response in* L. sordida

**Figure 5.**
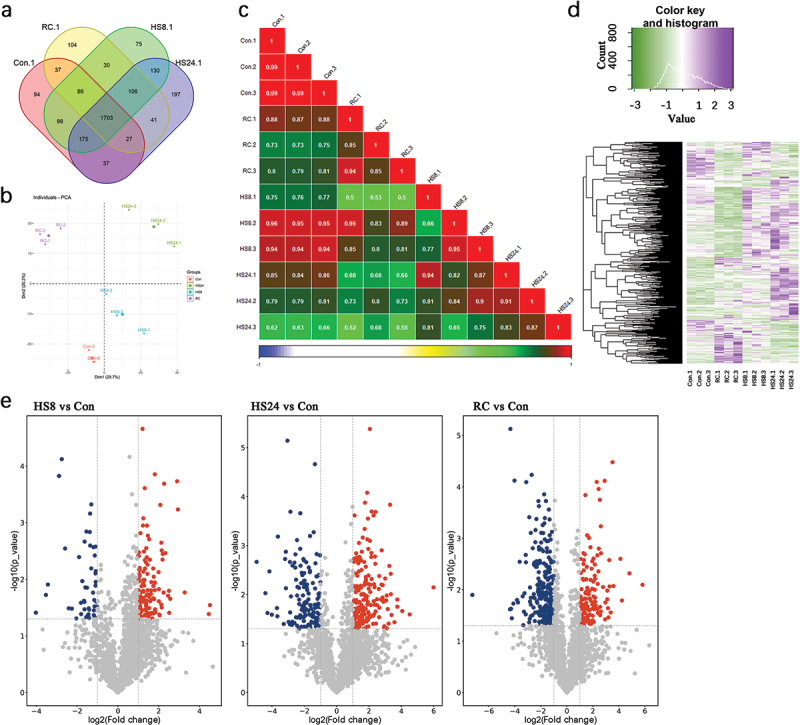
Comparative proteomic analysis of *Lepista sordida* mycelium under different conditions to study its response to heat stress. (a) Venn diagram of proteins identified in the proteomes of Con, HS8, HS24, and RC groups. (b) The expression profiles of the samples were visualized by PCA analysis. (c) Pearson correlation analysis was performed for the expression of genes identified in all four groups of the twelve samples. (d) Heat map created from 974 proteins identified in all samples by hierarchical clustering using paired euclidean distance. (e) Volcano plot showing the changes in protein expression in the Con, HS8, HS24, and RC groups.

To investigate the mechanisms of heat stress resistance of *L. sordida*, proteomic analyses of *L. sordida* mycelia were performed under different heat stress conditions. Proteins were extracted from mycelia of the Con group, HS8 group, HS24 group, and RC group as described in Section 2.5 (Supplementary Figure S1) and subjected to label-free quantitative proteomic analysis. A total of 2,940 proteins were identified (Figure 5a and Supplementary Table S2). Among these, 1,703 proteins were common to all four groups, with 2,257, 2,403, 2,416, and 2,134 proteins specifically identified in the Con group, HS8 group, HS24 group, and RC group, respectively. Within the identified proteins, the Con group had 94 unique proteins, the HS8 group had 75 unique proteins, the HS24 group had 197 unique proteins, and the RC group had 104 unique proteins. In contrast to the Con and RC groups, the HS8 and HS24 groups together had 402 unique proteins (Figure 5a).

Principal component analysis (PCA) showed distinct differences among the four groups (Figure 5b), and Pearson correlation analysis indicated a high correlation (>0.65) among samples within the same group (Figure 5c). These results confirm the reliability of the data for the subsequent analysis. The subsequent clustering analysis using a heat map showed that the samples from each group clustered distinctly together. Notably, the HS8 group was closely associated with the HS24 group, while a division was observed between the RC group and the other three groups (Figure 7d).

Analysis of the proteomics of *L. sordida* revealed a multitude of differentially expressed proteins (DEPs) between the Con group and each of the different heat stress groups. A 2.0-fold change cut off and *p*-value < 0.05 were used to classify the differentially expressed proteins (DEPs). Compared to the Con group, a higher number of DEPs (207 upregulated and 157 downregulated) was observed in the HS24 group than in the HS8 group (138 upregulated and 53 downregulated). Interestingly, the RC group showed a higher number of downregulated proteins (255) than upregulated proteins (143) compared to the Con group (Figure 5e and Supplementary Table S3).

The DEPs between the Con group and the heat stress treated groups (HS8 or HS24) instead of the DEPs between the Con group and the RC group are crucial for understanding the mechanism of the heat stress response in *L. sordida*. A total of 2,836 proteins were identified in the Con, HS8, and HS24 groups, of which 579 proteins were detected in the heat stress treated groups (HS8 or HS24) but not in the Con group. In comparison to the Con group, 268 proteins showed significant upregulation (FC > 2 and *p*-value < 0.05) in the heat stress-treated groups (HS8 or HS24). These 847 proteins were designated as HS-up DEPs. Correspondingly, 131 proteins exclusively present in the Con group (compared to the HS8 and HS24 groups) and 167 proteins significantly downregulated in the heat stress-treated groups (HS8 or HS24) (FC < 0.5 and *p*-value < 0.05) were designated as HS-down DEPs (Supplementary Table S4).

**Figure 6.**
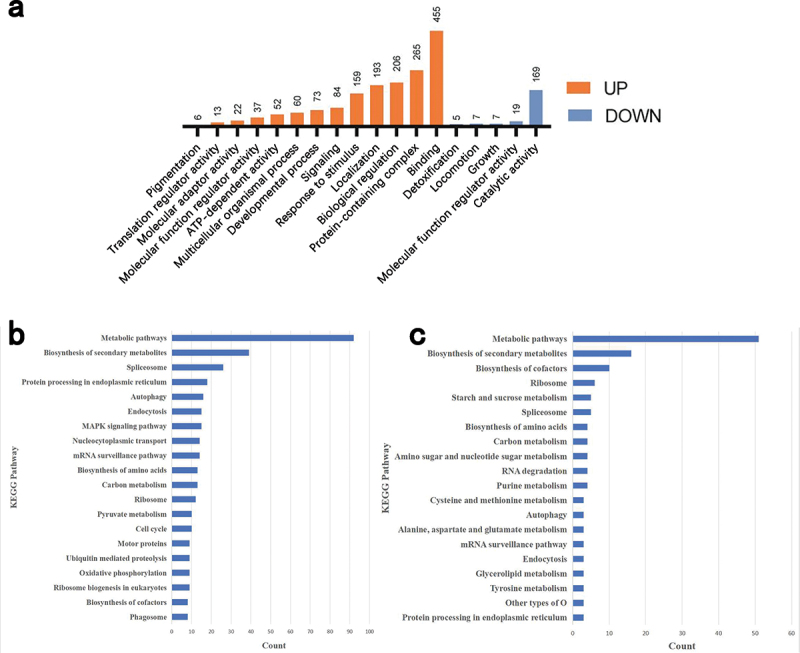
Enrichment analysis of differentially expressed proteins upon heat stress treatment. (a) Enriched GO terms of HS-up DEPs and HS-down DEPs. (b, c) KEGG pathways enriched in HS-up DEPs and HS-down DEPs; (b) the top 20 of KEGG enrichment in HS-up DEPs; (c) the top 20 of KEGG enrichment in HS-down DEPs.

To determine the functions of these DEPs, a Gene Ontology (GO) annotation enrichment analysis was performed for HS-up DEPs and HS-down DEPs (Figure 6a). The results of the enrichment analysis revealed the distinct distribution of proteins within HS-up DEPs and HS-down DEPs. Specifically, HS-up DEPs are primarily associated with processes such as binding, protein-containing complex, biological regulation, localisation and response to stimulus, whereas HS-down DEPs are mainly associated with processes such as catalytic activity, molecular function regulator activity, locomotion, growth and detoxification. KEGG pathway analysis was performed for the DEPs of each comparison (Figure 6b,c). Specifically, the enriched KEGG pathways in the HS-up DEPs are mainly distributed in the metabolic pathways, biosynthesis of secondary metabolites, spliceosome, protein processing in the endoplasmic reticulum and autophagy (Figure 6b). While the enriched KEGG pathways in the HS-down DEPs are mainly distributed in metabolic pathways, biosynthesis of secondary metabolites, biosynthesis of cofactors, ribosome, starch and sucrose metabolism (Figure 6c).

## 4. Discussion

*Lepista* *sordida* was first described in 1821 by Elias Magnus Fries under the genus *Agaricus* with the epithet *sordidus* (Thongbai et al. 2017). Currently, there are numerous synonyms of *L. sordida* documented in the GenBank database (*Collybia sordida*, TaxID: 123925), Index Fungorum (http://www.indexfungorum.org/) and MycoBank (http://www.mycobank.org/) databases, including *Gyrophila nuda* var. *lilacea* Quél. 1888, *L. nuda* var. *sordida* (Schumach.) Maire 1916, *Rhodopaxillus sordidus* var. *aianthinus* Bon 1970, and others (Thongbai et al. 2017). In addition, the genus *Lepista* is a polyphyletic genus with over 50 species. Some species within the genus are difficult to distinguish due to the similarity of their fruiting bodies (Alvarado et al. 2017). The *in silico* DNA-DNA hybridisation (*is*DDH), which relies on whole genome sequences, has become the gold standard for genomically circumscribing species in current prokaryotic taxonomy (Richter et al. 2009). However, there is little genome-based evolutionary and taxonomic data for *L. sordida*. In this study, genome sequencing was performed to investigate the evolutionary status and genetic information of functional genes of *L. sordida*. Genome-based similarity indexes including *is*DDH, ANI, phylogenomic tree analysis, and collinearity analysis were performed. The ANI and *is*DDH percentages between monokaryotic mycelia Lds1 and Lds5 derived from the same dikaryotic strain *L. sordida* WT2342 were 99.02% and 90.80%, respectively. The results indicated that the ANI and *is*DDH percentages between the monokaryotic mycelia of species *L. sordida* are higher than 99.00% and 90.00%, respectively. The ANI (threshold value: 95%–96%) and *is*DDH (threshold value: 70%) percentages were genomic nucleic acid-level comparisons utilised for delineating prokaryotic species (Chun et al. 2018). However, until now, there are no defined ANI and *is*DDH threshold values for fungal species classification. Based on the phylogenetic and collinearity analysis, the phylogenetic relationship between *L. sordida* and other commercially cultivated edible mushrooms, likely served as a reference model for the cultivation and breeding of *L. sordida* during large-scale industrialisation.

Substrate utilisation and environmental factors are crucial determinants for the successful fruiting of edible mushrooms. CAZymes analysis revealed the presence of a full repertoire of polysaccharide-degrading enzymes in *L. sordida* (Figure 3). Wild *L. sordida* strains typically live in grasslands, suggesting that the nutrient requirements of *L. sordida* are similar to those of straw-rotting mushrooms, such as *A. bisporus*. Genome analysis of *A. bisporus* has shown that the distribution of lignin-depolymerisation enzymes, such as hemi-thiolate peroxidases and *β*-etherases, differs from those of the wood-decaying fungi, suggesting that these enzymes may be related to its adaptation to the humic-rich niche (Morin et al. 2012). To further analyse the nutritional requirements of *L. sordida*, the proteins of the AA1 and AA2 families were compared with other 8 edible and medicinal mushrooms (Supplementary Figures S6, S7). The genome of *L. sordida* encodes 16 genes of the AA1 family and 5 genes of the AA2 family. The number of AA2 family genes is lower than in most wood-rotting mushrooms. According to the studies by Patyshakuliyeva et al. (2015), the most highly expressed ligninolytic proteins are two AA1 laccases (EKM75207 and EKM75206) and an AA1 manganese peroxidase (EKM80806), suggesting that their pivotal role in compost substrate utilisation. Phylogenetic analysis (Supplementary Figures S6, S7) revealed that the closest relatives of EKM75207, EKM75206, and EKM80806 are all from *L. sordida* (MDBLsor1_01908 and MDBLsor1_04905), implying similarities in substrate selectivity between *A. bisporus* and *L. sordida*. The results confirmed that humic-rich compost should be used as a substrate for the production of *L. sordida*.

High temperatures have a significant impact on the yield and quality of mushrooms (Albataineh and Kadosh 2016), and *L. sordida* is no exception. The wild strains of *L. sordida* in Sandaoguan Nature Reserve died out due to the elevated temperatures in the autumn of 2010 (Zheng et al. 2011). In addition, temperatures above 34 °C inhibited the mycelial growth of strain *L. sordida* (Xie and Hu 2005; Zhang et al. 2006). In the present study, it was also observed that the mycelium of *L. sordida* WT2342 could not grow under conditions higher than 34 °C. In real mushroom production, mycelia are usually not exposed to high temperatures for a prolonged period. In most cases, they are exposed to short-term heat stress resulting from factors such as heat dissipation from mycelial growth, sudden increases in ambient temperature and inadequate ventilation for cooling (Xu et al. 2021). Therefore, the ability of mycelia to withstand short-term heat stress is crucial for successful production. Understanding the response mechanism of *L. sordida* to heat stress is crucial for breeding high-quality strains suitable for industrial cultivation. In this study, a label-free comparative proteomic analysis was performed to reveal the response to heat stress in *L. sordida*.

Heat shock proteins (HSPs), which act as molecular chaperones, play pivotal roles in the cellular response to heat stress and contribute significantly to thermotolerance (Wang et al. 2018). The leading class of HSPs comprises five major families that are highly conserved across different species: these include the HSP100s, HSP90s, HSP70s, HSP60s, and the small heat shock proteins, known as sHSPs (Richter et al. 2010; Xu et al. 2021). In this study, nine sHSPs, two HSP60s, four HSP70s, one HSP90, and one HSP100 were identified in HS-up DEPs (Table 5 and Supplementary Table S4).

**Table 5.** Differentially expressed proteins identified in the proteomic data under heat treatment.

ID	Functional description	Fold change		
		HS24/Con	HS8/Con	RC/Con
**sHSPs:**				
MDBLsor1_02090	16.9 kDa class I heat shock protein 3	28.98	21.94	0
MDBLsor1_06009	DnaJ protein homolog ANJ1	2.76	1.76	1.7
MDBLsor1_03925	Heat shock protein homolog C338.06c	∞	∞	∞
MDBLsor1_03916	Heat shock protein 16	∞	∞	∞
MDBLsor1_03917	17.8 kDa class I heat shock protein	∞	∞	∞
MDBLsor1_03920	Heat shock protein 16	∞	∞	∞
MDBLsor1_03921	17.8 kDa class I heat shock protein	∞	∞	∞
MDBLsor1_03946	17.8 kDa class I heat shock protein	∞	∞	∞
20MDBLsor1_03947	Heat shock protein 16	∞	∞	∞
**HSP60s:**				
MDBLsor1_00439	Heat shock protein sti1 homolog	7.28	3.50	0.89
MDBLsor1_10863	Heat shock protein 60	2.26	1.41	0.6
**HSP70s:**				
MDBLsor1_05698	Heat shock protein HSS1	5.30	3.50	1.24
MDBLsor1_05119	Heat shock protein hsp88	2.37	1.38	0.91
MDBLsor1_04438	Heat shock 70 kDa protein	2.89	2.02	0.54
MDBLsor1_11763	Heat shock protein 78	∞	∞	∞
**HSP90s:**				
MDBLsor1_06083	Heat shock protein 90 homolog	7.99	3.54	0.41
**HSP100s:**				
MDBLsor1_03253	Heat shock protein 104	63.59	22.82	11.95
**Others:**				
MDBLsor1_00641	Heat shock transcription factor	∞	NA	NA
MDBLsor1_05908	Ubiquitin-conjugating enzyme E2 C	∞	NA	NA
MDBLsor1_05401	E3 ubiquitin-protein ligase hel	∞	NA	NA
MDBLsor1_00616	Proteasome subunit alpha type-3	2.64	1.91	2.08
MDBLsor1_10649	Autophagy-related protein 18	∞	∞	∞
MDBLsor1_09390	Calmodulin	∞	∞	∞
MDBLsor1_07667	Calcium-transporting ATPase 2	∞	∞	NA
MDBLsor1_06126	Calcium pump	∞	∞	NA
MDBLsor1_00497	Vacuolar calcium ion transporter	20.67	6.02	5.43
MDBLsor1_01014	Calcium channel YVC1	3.86	3.93	6.09
MDBLsor1_11430	Mitogen-activated protein kinase mpkA	∞	∞	∞
MDBLsor1_07096	Thioredoxin reductase	4.22	2.37	3.93
MDBLsor1_09192	Thioredoxin peroxidase	∞	∞	∞
MDBLsor1_00247	Thioredoxin-like protein AAED1	∞	∞	∞

∞ means that the specific protein was not detected in the Con group, but was detected in the HS24 or HS8 or RC group. NA stands for missing values.

The small heat shock proteins (sHSPs) form a widespread and diverse group of molecular chaperones. The molecular weight of sHSPs ranges from 12 to 43 kilodaltons (kDa) (Haslbeck et al. 2005). sHSPs bind to partially denatured proteins and thus prevent the irreversible inactivation and aggregation of proteins during heat stress (Haslbeck et al. 2005; Mahmood et al. 2010; Richter et al. 2010; Wang et al. 2018a). Here, one sHSP (MDBLsor1_02090) showed significant upregulation, namely a 28.98-fold increase in the HS24 group and a 21.94-fold increase in the HS8 group compared to the Con group, and then returned to normal levels after recovery cultivation (Table 5). In addition, seven sHSPs were detected in the heat stress-treated groups (HS8 and HS24), which were not present in the Con group. It has been reported that some members of the sHSP family are either inactive or only partially active under normal physiological conditions. However, when exposed to heat shock, they switch to an active state. Once activated, sHSPs are able to bind to denatured proteins and form a stable sHSP-substrate complex. This interaction effectively prevents the aggregation of proteins from becoming irreversible (Haslbeck et al. 2005). sHSPs in *L. sordida* could protect proteins from irreversible aggregation under heat stress.

Members of the HSP60s family are constitutively expressed and fulfil essential functions as molecular chaperones that facilitate protein folding under normal cellular conditions (Langer and Neupert 1990; Martin et al. 1992). However, the concentration of yeast in the mitochondrial HSP60 increases two- to threefold at 42 °C (Martin et al. 1992). In this study, two HSP60s (MDBLsor1_00439 and MDBLsor1_10863) were upregulated under heat stress and then returned to normal levels after recovery culture. Similar results were also observed in *Pleurotus ostreatus* and *Hypsizygus marmoreus* (Xu et al. 2021). This induction could be due to an increased requirement for these HSP60s to protect pre-existing proteins from denaturation under heat stress (Martin et al. 1992). HSP60s are also known as mitochondrial chaperones, which can stabilise the inner and outer membranes of mitochondria and prevent cell apoptosis (Tang et al. 2018). Based on previous studies (Martin et al. 1992; Tang et al. 2018), members of the HSP60s family could stabilise pre-existing proteins under heat stress and stabilise the inner and outer membranes of mitochondria and prevent cell apoptosis in *L. sordida*.

HSP70s, the most conserved heat shock proteins, are thought to be cytoprotective and essential for the development of thermotolerance (Sharma et al. 2007). The expression of HSP70 genes in *Lentinula edodes* and *Pleurotus ostreatus* was upregulated under heat stress (Fu et al. 2016; Wang et al. 2018a; Zou et al. 2018). In our previous report, an HSP70 (A0A369K2K3) gene, *hmHsp70*, was cloned from *H. marmoreus* and cloned into tobacco. The transgenic tobacco showed increased resistance to lethal temperatures (Xu et al. 2020). Here, one HSP70s (MDBLsor1_11763) was detected in the heat stress-treated groups, which was not present in the Con group, and three other HSP70s (MDBLsor1_05698, MDBLsor1_05119, and MDBLsor1_04438) were upregulated under heat stress. HSP70s could confer thermotolerance to *L. sordida* during heat shock. In addition, HSP70s could also help in the release of substrate proteins from the sHSPs-substrate complex, emphasising their cooperative function with sHSPs (Haslbeck et al. 2005).

HSP90s have diverse functions in cellular processes such as signal transduction networks, cell cycle regulation, protein degradation, and protein trafficking (Yamada et al. 2007; Mahmood et al. 2010). One of its main functions is that of a molecular chaperone, which plays a crucial role in preventing protein aggregation and facilitating refolding of denatured proteins (Yonehara et al. 1996). Studies have shown that HSP90s play an important role in preventing the aggregation of unfolded proteins under stress conditions in yeast (Richter et al. 2010). In this study, the expression of an HSP90 (MDBLsor1_06083) increased threefold after exposure to heat shock at 37 °C for 8 hours and even more than sevenfold after 24 hours. This finding confirmed the involvement of HSP90 in the cellular response to heat stress in *L. sordida*, probably by preventing protein aggregation and supporting refolding of denatured proteins.

Proteins of the HSP100s family generally have the task of protecting proteins from denaturation and aggregation (Wang et al. 2004). The *HSP104* mutant strain of *Saccharomyces cerevisiae* was not thermotolerant. However, the introduction of the wild-type gene into the mutant cells successfully restored their thermotolerance (Sanchez and Lindquist 1990). HSP104 is induced by heat and can reactivate denatured proteins, thereby improving cell survival after extreme heat exposure in *S. cerevisiae* (Glover and Lindquist 1998). Furthermore, the thermotolerance defect in the *S. cerevisiae Hsp104* mutant could also be complemented by the overexpression of an *HSP100* gene, *PsHSP100*, from *Pleurotus sajor-caju* (Lee et al. 2006). These results suggest that HSP104 plays a crucial role in cell survival at extreme temperatures. Members of the HSP100s family are upregulated by environmental stress (Mahmood et al. 2010). In this study, one HSP104 (MDBLsor1_03253) showed a 22.82-fold upregulation in the HS8 group, a 63.59-fold upregulation in the HS24 group, and an 11.95-fold upregulation in the RC group compared to the Con group. HSP104 in *L. sordida* could protect against protein denaturation and thereby improve cell survival under heat stress.

**Figure 7.**
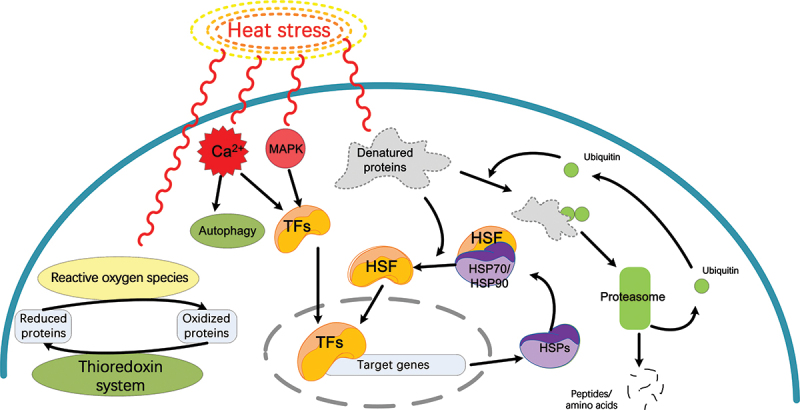
Schematic model for the role of signalling pathways, HSF, HSPs, the ubiquitin-proteasome, and the thioredoxin system in the response to heat stress in *Lepista sordida*. The schematic model was visualised with Visio.

High expression of heat shock proteins is ubiquitous in mushrooms’ resistance to high temperature stress, such as *L. edodes* (Zhang et al. 2023), *H. marmoreus* (Xu et al. 2021), *Pleurotus tuoliensis* (Chen et al. 2023), *Ganoderma lucidum* (Liu et al. 2018a), *Pleurotus ostreatus* (Zou et al. 2018), *Grifola frondosa* (Xie et al. 2024), *Flammulina filiformis* (Liu et al. 2020), and *Wolfi-poria cocos* (Hu et al. 2023). Overall, it is hypothesised that the accumulation of heat shock proteins plays a crucial role in the response to heat stress in *L. sordida* (Figure 7).

Heat stress transcription factors (HSFs) play a crucial role by coordinating different Hsf members and interacting with chaperones to regulate gene expression at the transcriptional level, thereby increasing tolerance to heat stress (Song et al. 2013). The detection of a single HSF (MDBLsor1_00641) exclusively in the HS24 group indicates that the amount of this particular HSF (MDBLsor1_00641) increases specifically under severe heat stress conditions. This indicates the crucial role of HSF in high-temperature and prolonged heat stress environments (Figure 7).

The ubiquitin-proteasome system is a widespread protein remodelling system that can degrade or remove abnormal and short-lived proteins in eukaryotic cells. Originally, abnormal and short-lived proteins were tagged with ubiquitin to mark them for degradation by the proteasome. Subsequently, these targeted proteins are degraded by the proteasome and finally, the ubiquitin molecules are recycled for further ubiquitination cycles (Liu et al. 2020a). In this system, the ubiquitin-conjugating enzyme (E2) and the ubiquitin-ligase enzymes (E3) play a crucial role in the covalent binding of ubiquitin to specific target proteins (Liu et al. 2020a). In the current study, the ubiquitin-conjugating enzyme E2 C (MDBLsor1_05908) and the E3 ubiquitin-protein ligase hel (MDBLsor1_05401) were identified in the HS24 group, while the proteasome subunit alpha type-3 (MDBLsor1_00616) was upregulated under heat stress. Previous reports showed upregulation of the proteasome or its subunits under heat stress in *Pleurotus tuberregium* or in *H. marmoreus*, respectively (Huang et al. 2010; Xu et al. 2021). These results suggest that the ubiquitin-proteasome system likely plays a key role in the removal of denatured proteins produced during heat stress (Figure 7).

To investigate the morphological changes in response to heat stress, the mycelia were examined under a positive fluorescence microscope after staining with Congo red and DAPI. The images showed that the nuclei were fragmented and dispersed throughout the mycelia when exposed to 37 °C for 24 hours. The fragmentation of the nuclei is considered a morphological indicator of autophagy. In addition, morphological features characteristic of apoptosis, such as nuclear condensation, accumulation of reactive oxygen species, and DNA fragmentation, were observed in two *Pleurotus* species exposed to heat stress (Song et al. 2014). Xu et al. (2021) reported nuclear fragmentation in hyphal cells as an indication of heat stress-induced apoptosis in *H. marmoreus* (white variety). In addition, an autophagy-related protein 18 (MDBLsor1_10649) was identified in the heat stress groups (HS8, HS24, and RC groups), which was absent in the Con group. The catabolic process of autophagy is dependent on autophagy-related proteins (ATGs) (Bansal et al. 2017). In most cases, mushroom mycelia were able to resume growth after exposure to a high temperature stress of 37 °C, albeit only after a recovery period of several days. Some mushroom mycelia were also able to resume growth after exposure to 40 °C, but required a longer time. High temperature-induced autophagy and nucleosome fragmentation might be among the primary factors contributing to the cessation of mycelial growth. However, this process might also provide nutrients to other mycelia and thus represent a strategy to ensure the survival of these mycelia.

The signal transduction pathway plays a significant role in sensing high temperatures and adjusting cellular metabolism and function to prevent heat-related damage (Mittler et al. 2012). Ca^2+^ is involved in heat shock signal transduction and regulates downstream events in filamentous fungi (Zhang et al. 2016). Liu et al. (2018b) reported that NO and Ca^2+^ signals mutually promoted each other in response to heat stress in *G. lucidum*, potentially regulating the synthesis of ganoderic acid. Calmodulin (MDBLsor1_09390) was detected in heat stress groups (including HS8 group, HS24 group, and RC group) in *L. sordida*. Calmodulin, a small Ca^2+^-binding protein, is involved in crucial cellular pathways by regulating the function of other proteins (Alaimo and Villarroel 2018). Calcium-transporting ATPase 2 (MDBLsor1_07667) and the calcium pump (also known as Ca^2+^-ATPase or SERCA) (MDBLsor1_06126) are expressed in the HS8 group and the HS24 group and contribute to the intracellular transport of calcium ions (Brini 2009; Primeau et al. 2018). The vacuolar calcium ion transporter (MDBLsor1_00497) and the calcium channel YVC1 (MDBLsor1_01014) were significantly upregulated in the RC group (5.43-fold and 6.09-fold upregulation, respectively). The results suggest that the Ca^2+^ signal may play a central role in heat stress signal transduction in *L. sordida* (Figure 7). In addition, mitogen-activated protein kinase (MAPK) signalling pathways play a crucial role in enabling eukaryotic cells to adapt to environmental stress. The study of Xu et al. (2021) revealed that MAPK cascade is involved in heat stress signal transduction in *H. marmoreus*. Here, four mitogen-activated protein kinases, one MAP kinase kinase and one MAP kinase kinase kinase were identified in the proteomic data. One of them (MDBLsor1_11430) was a unique protein among all heat groups, while the change of the other proteins showed no significant difference. It suggested that the MAPK cascade might be involved in the heat stress signal transduction (Figure 7).

Endogenous or exogenous protective agents could increase the thermotolerance of organisms. Among these agents, trehalose and ROS scavenging have been identified as protective agents against heat stress in mushrooms (Xie et al. 2024). Trehalose, a non-reducing disaccharide, is a compatible solute that protected superoxide dismutase (SOD) activity slightly and plays a direct role in the removal of H_2_O_2_ and O_2_^−^ in wheat under heat stress (Luo et al. 2008). It has been reported that the accumulation of trehalose and the upregulation of trehalose synthesis genes upon high-temperature treatment in different mushrooms (Lei et al. 2017; Liu et al. 2019; Xu et al. 2021). Four proteins related to trehalose synthesis were identified in the proteomic data, including trehalose phosphorylase and trehalose-phosphate synthase, but no clear trend of change was observed upon heat treatments. Similar results were observed for catalase (CAT), peroxidase (POX), and superoxide dismutase (SOD). However, a thioredoxin reductase (MDBLsor1_07096) was significantly up-regulated in the HS24, HS8, and RC groups, one thioredoxin peroxidase (MDBLsor1_09192) and the thioredoxin-like protein AAED1 (MDBLsor1_00247) were identified in HS24, HS8, and RC groups. The thioredoxin system in *Saccharomyces cerevisiae* serves as a defence mechanism against oxidative stress. Components such as thioredoxin, thioredoxin reductase, and thioredoxin peroxidase, involved in this system can be upregulated in response to exposure to reactive oxygen species (ROS) (Morano et al. 2012). These results suggested that the thioredoxin system, rather than trehalose synthesis, catalase, peroxidase, and superoxide dismutase, might be involved in response to the heat stress in *L. sordida* (Figure 7).

## 5. Conclusions

In conclusion, the high-quality genome of strain *L. sordida* was sequenced using Nanopore and Illumina sequencing platforms. The genomic comparative and evolutionary analysis has significantly enriched our understanding of the taxonomic classification of *L. sordida*. Proteomic analysis was performed to explore the mechanisms underlying mycelial response to heat stress in *L. sordida*. Experimental studies and data analysis of mycelia subjected to various treatments confirmed increased expression of proteins associated with protein folding and degradation, signal transduction and the thioredoxin system, which together protect cells from heat stress. The distinctive characteristics of *L. sordida* in response to heat stress could facilitate a deeper exploration of the molecular mechanisms underlying thermotolerance.

## Supplementary Material

Supplemental Material

## Data Availability

This whole genome shotgun project of strain Lds1 has been deposited at DDBJ/ENA/GenBank under the accession JAYKXK000000000. The version described in this paper is version JAYKXK010000000. This whole genome shotgun project of strain Lds5 has been deposited at DDBJ/ENA/GenBank under the accession JAYKYZ000000000. The version described in this paper is version JAYKYZ010000000. The raw data for the proteome analysis reported in this paper have been deposited in the OMIX, China National Center for Bioinformation/Beijing Institute of Genomics, Chinese Academy of Science under accession number OMIX005380. The genome sequences and other information of this paper are also available at https://file.mushroomlab.cn.
