# Supplementary material for: Insights into the evolution and mechanisms of response to heat stress by whole genome sequencing and comparative proteomics analysis of the domesticated edible mushroom Lepista sordida
Source: Mycology. 2024 Jul 7;16(1):324–43. doi: 10.1080/21501203.2024.2363620 (PMC11899243; doi:10.1080/21501203.2024.2363620)
Supplement: Supplemental Material [file TMYC_A_2363620_SM5154.zip › Supplementary_figures_and_Table_S1.docx]

# Insights into the evolution and mechanisms of response to heat stress by whole genome sequencing and comparative proteomics analysis of the domesticated edible mushroom *Lepista sordida*

Yanjiao Zhang^§1^, Chengzhi Mao^§1^, Xuyang Liu^1^, Lizhong Guo^1^, Chunhui Hu^1^, Xiaobo Li^2^, Lili Xu*^1^, Hao Yu*^1^

^1^Shandong Provincial Key Laboratory of Applied Mycology, School of Life Sciences, Qingdao Agricultural University, 700 Changcheng Road, Chengyang District, Qingdao 266109, Shandong Province, PR China. ^2^Shandong Mushroom Industrial Technology Innovation Research Institute.

Running title: Evolutionary patterns and heat stress responses of *Lepista sordida*

§Yanjiao Zhang and Chengzhi Mao contributed equally to this work.

*Corresponding author:

Hao Yu E-mail: yuhaosunshine@163.com

Lili Xu E-mail: ellyxu@163.com

Mailing address: 700 Changcheng Road, Chengyang District, Qingdao, Shandong Province, People’s Republic of China

**Keywords:** edible mushroom; *Lepista sordida*; genome; proteome; heat stress response

**Table S1. Genome assembly features of monokaryotic *Lepista sordida* Lds5.**

| **Characteristics** | ***Lepista sordida* Lds1** |
| --- | --- |
| Genome assembly size (Mb) | 35.57 |
| Contigs | 1511 |
| Longest Contigs (kb) | 553.6 |
| Contigs N50 (kb) | 78.4 |
| Contigs N90 (kb) | 12.5 |
| GC (%) | 45.90 |
| Sequencing methods | Illumina |

**Figure S1.**

**
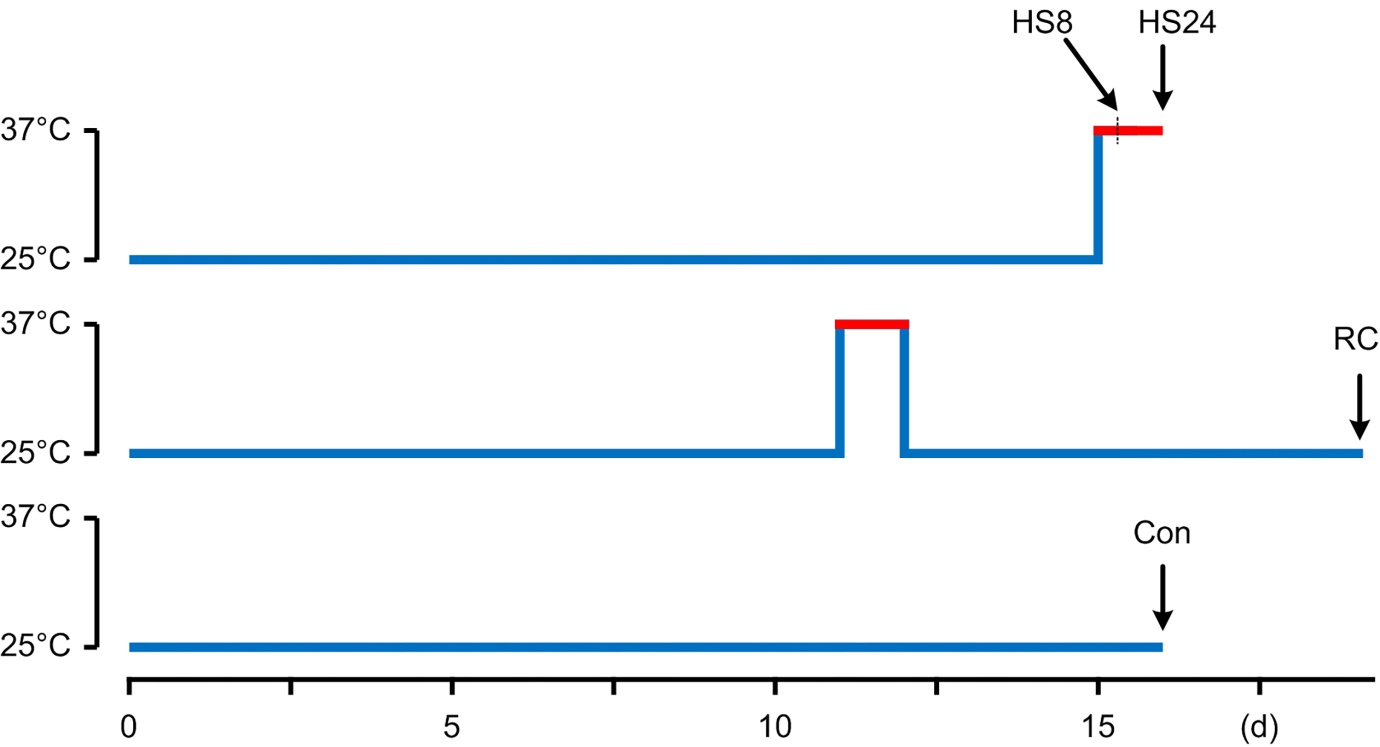
**

**Figure S1.** Cultivation conditions for different groups. The mycelia were cultivatedon YMA plates at 25 °C. Heat stress was definedas exposure at 37 °C.

**Figure S2.**

**
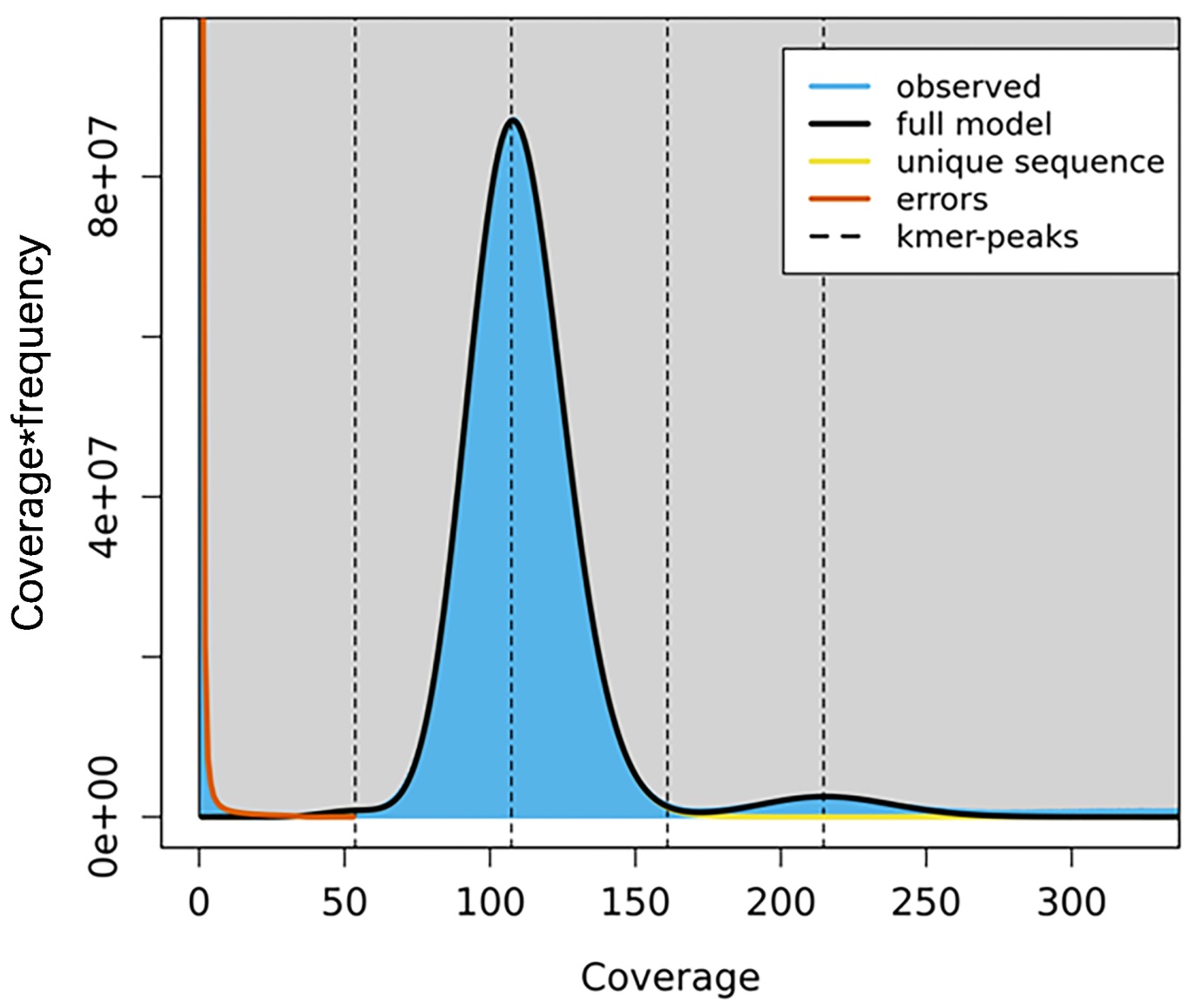
**

**Figure S2.** Histogram of the 19-mer depth distribution of Illumina sequencing reads of *Lepistasordida* Lds1 displayed in GenomeScope. The blue areas show the observed k-mer frequencies and the black line show the fitted GenomeScope model.

**Figure S3.**

**
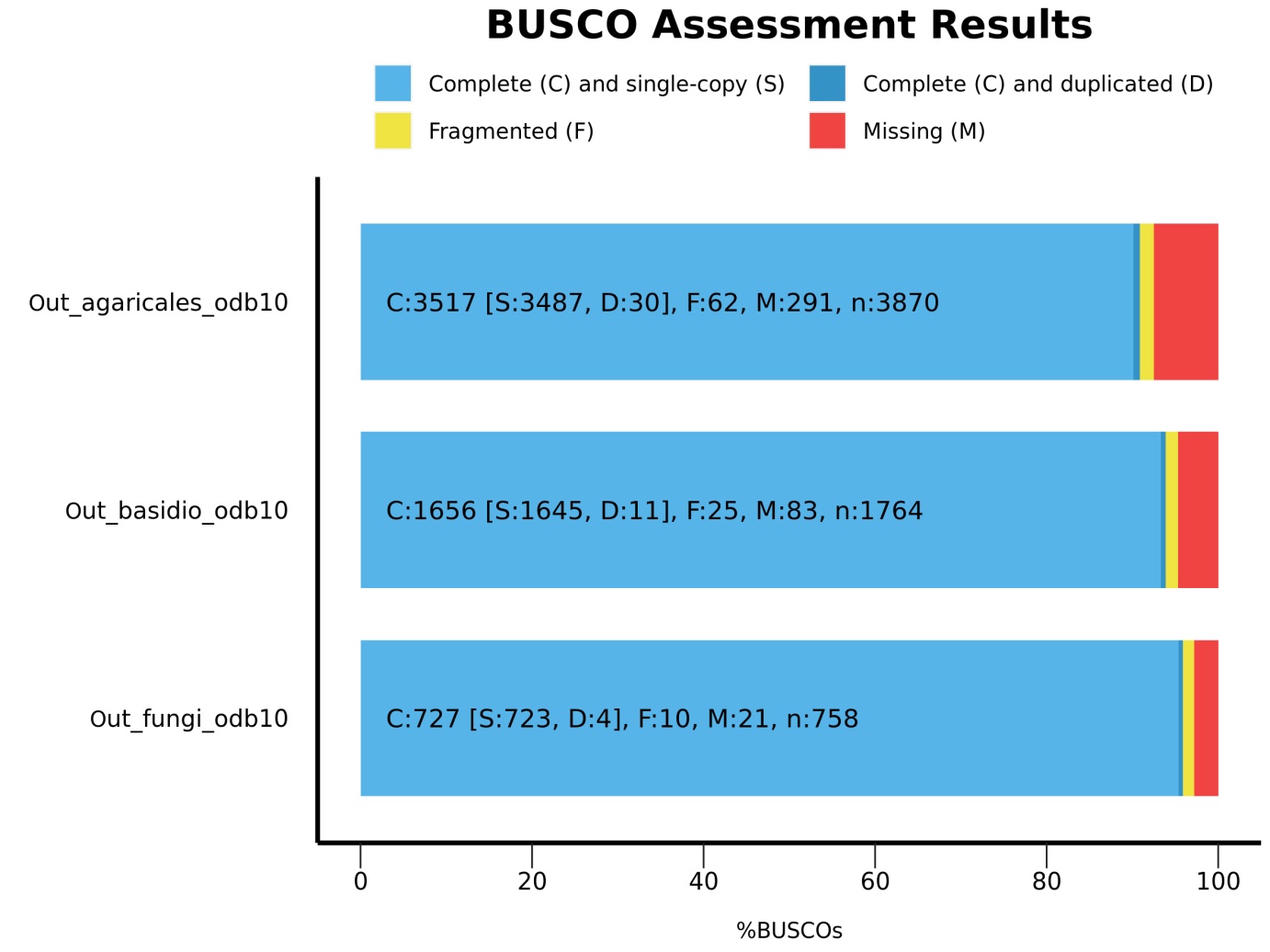
**

**Figure S3.** BUSCO assessment of protein annotation completeness in *Lepista sordida* Lds1 using different BUSCO databases. The completeness of gene prediction was assessedusing the databases fungi_odb10 (95.9%), basidiomycota_odb10 (93.9%), and agaricales_odb10 (90.9%), respectively.

**Figure S4.**

**
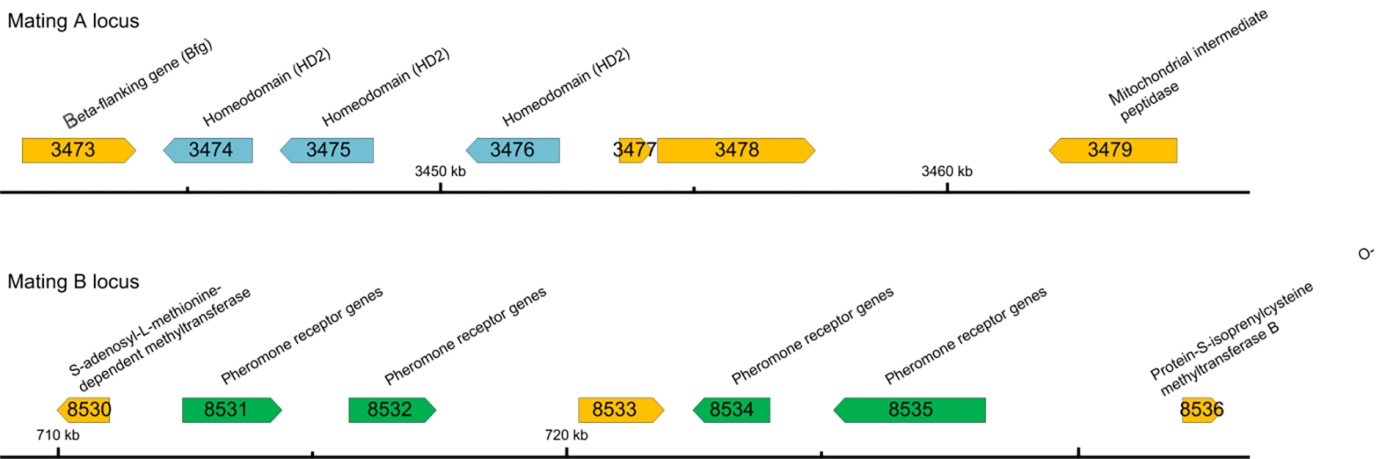
**

**Figure S4.** Gene structure of the mating type loci of *Lepista sordida*.

**Figure S5.**

**
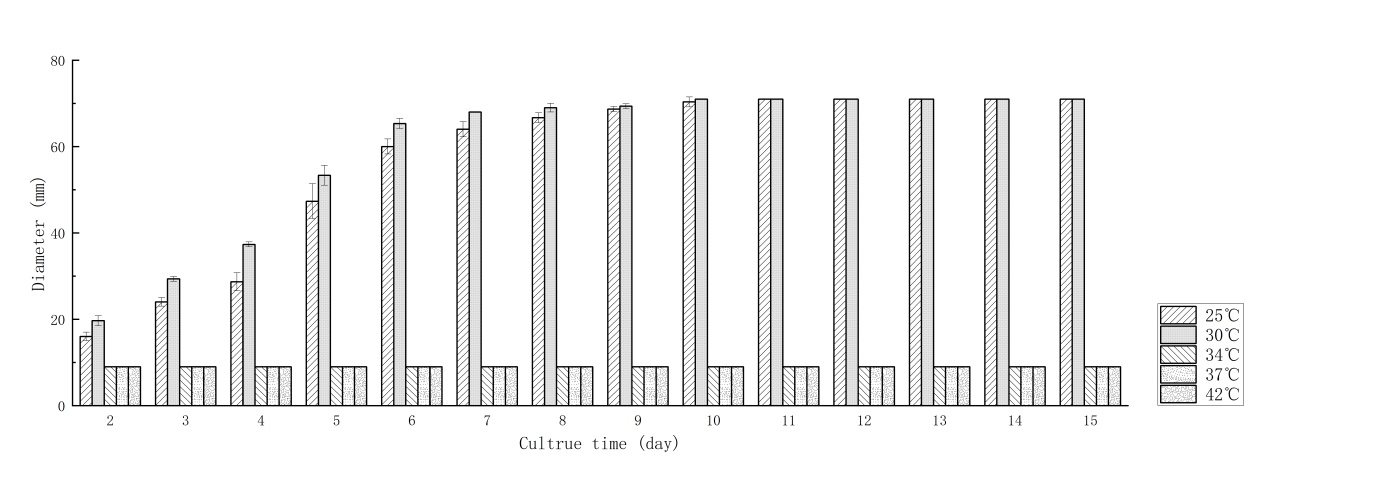
**

**Figure S5.** Growth at different temperatures (25, 30, 34, 37 and 42 °C) of strain *Lepista sordida* on YMA plates for 15 days.

**Figure S6.**

**
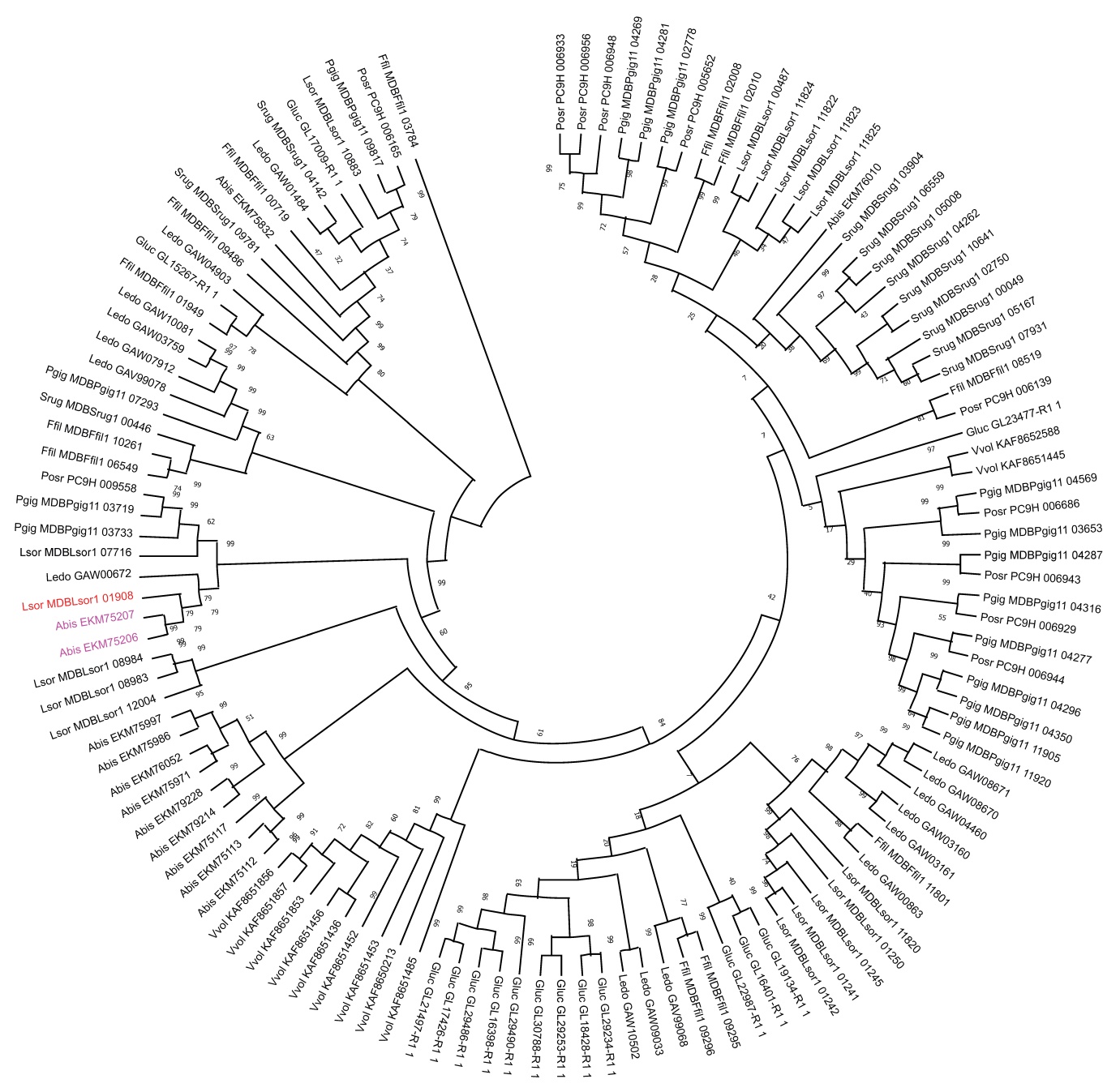
**

**Figure S6.** Phylogenetic analysis of the laccases proteins of *Lepista sordida* (from AA1 CAZymes family) and the laccases of other 8 edible mushrooms. Abis: *Agaricus bisporus* var. burnettii JB137-S8; Ffil: *Flammulina filiformis* Fv1-10; Gluc: *Ganoderma lucidum* G.260125-1; Ledo: *Lentinula edodes* NBRC 111202; Lsor, *L. sordida* Lds1; Pgig: *Pleurotus giganteus*; Posr: *Pleurotus ostreatus* PC9; Srug: *Stropharia rugosoannulata*; Vvol: *Volvariella volvacea*.

**Figure S7.**

**
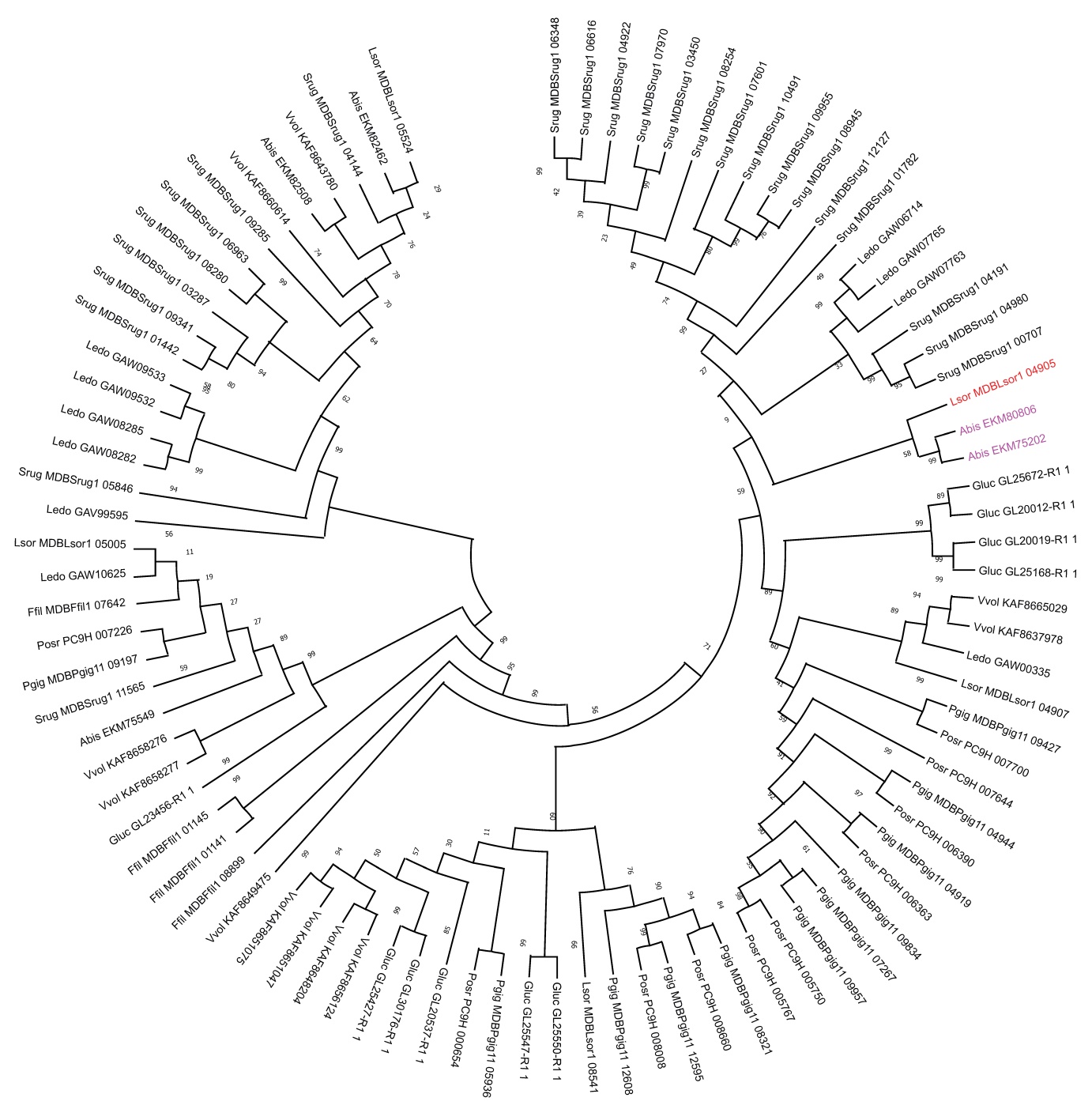
**

**Figure S7.** Phylogenetic analysis of the peroxidases proteins of *Lepista sordida* (from AA2 CAZymes family) and the peroxidases of other 8 edible mushrooms. Abis: *Agaricus bisporus* var. burnettii JB137-S8; Ffil: *Flammulina filiformis* Fv1-10; Gluc: *Ganoderma lucidum* G.260125-1; Ledo: *Lentinula edodes* NBRC 111202; Lsor, *L. sordida* Lds1; Pgig: *Pleurotus giganteus*; Posr: *Pleurotus ostreatus* PC9; Srug: *Stropharia rugosoannulata*; Vvol: *Volvariella volvacea*.
